# Investigating selected host and parasite factors potentially impacting upon seasonal malaria chemoprevention in Bama, Burkina Faso

**DOI:** 10.1186/s12936-020-03311-8

**Published:** 2020-07-06

**Authors:** Fabrice A. Somé, Thomas Bazié, Hanna Y. Ehrlich, Justin Goodwin, Aine Lehane, Catherine Neya, Kabré Zachari, Martina Wade, Jean-Marie Ouattara, Brian D. Foy, Roch K. Dabiré, Sunil Parikh, Jean-Bosco Ouédraogo

**Affiliations:** 1 Institut de Recherche en Sciences de la Santé, Direction Régionale de l’Ouest, 399 Avenue de la Liberté, 01, BP 545 Bobo-Dioulasso 01, Burkina Faso; 2 Yale Schools of Public Health and Medicine, Laboratory of Epidemiology and Public Health, 60 College Street, Room 724, New Haven, CT 06520 USA; 3 Arthropod-borne and Infectious Diseases Laboratory, Department of Microbiology, Immunology and Pathology, Colorado State University, Fort Collins, CO USA

**Keywords:** Seasonal malaria chemoprevention, *Plasmodium falciparum*, Microscopic and submicroscopic, Burkina Faso

## Abstract

**Background:**

Since 2014, seasonal malaria chemoprevention (SMC) with amodiaquine–sulfadoxine–pyrimethamine (AQ–SP) has been implemented on a large scale during the high malaria transmission season in Burkina Faso. This paper reports the prevalence of microscopic and submicroscopic malaria infection at the outset and after the first round of SMC in children under 5 years old in Bama, Burkina Faso, as well as host and parasite factors involved in mediating the efficacy and tolerability of SMC.

**Methods:**

Two sequential cross-sectional surveys were conducted in late July and August 2017 during the first month of SMC in a rural area in southwest Burkina Faso. Blood smears and dried blood spots were collected from 106 to 93 children under five, respectively, at the start of SMC and again 3 weeks later. Malaria infection was detected by microscopy and by PCR from dried blood spots. For all children, day 7 plasma concentrations of desethylamodiaquine (DEAQ) were measured and CYP2C8 genetic variants influencing AQ metabolism were genotyped. Samples were additionally genotyped for *pfcrt* K76T and *pfmdr1* N86Y, molecular markers associated with reduced amodiaquine susceptibility.

**Results:**

2.8% (3/106) of children were positive for *Plasmodium falciparum* infection by microscopy and 13.2% (14/106) by nested PCR within 2 days of SMC administration. Three weeks after SMC administration, in the same households, 4.3% (4/93) of samples were positive by microscopy and 14.0% (13/93) by PCR (p = 0.0007). CYP2C8*2, associated with impaired amodiaquine metabolism, was common with an allelic frequency of 17.1% (95% CI  10.0–24.2). Day 7 concentration of DEAQ ranged from 0.48 to 362.80 ng/mL with a median concentration of 56.34 ng/mL. *Pfmdr1* N86 predominated at both time points, whilst a non-significant trend towards a higher prevalence of *pfcrt* 76T was seen at week 3.

**Conclusion:**

This study showed a moderate prevalence of low-level malaria parasitaemia in children 3 weeks following SMC during the first month of administration. Day 7 concentrations of the active DEAQ metabolite varied widely, likely reflecting variability in adherence and possibly metabolism. These findings highlight factors that may contribute to the effectiveness of SMC in children in a high transmission setting.

## Background

In Burkina Faso, malaria is still the leading cause of morbidity and mortality with 7875,575 cases and 12,725 deaths in 2018 [1]. With these statistics, Burkina Faso has one of the highest malaria incidence rates in the world, and is one ‘high burden to high impact’ countries of the World Health Organization (WHO). In Burkina, malaria is highly seasonal with peak transmission occurring during the rainy season (June-October). Since 2005, the country has adopted several malaria control strategies to reduce the burden of the disease, including the provision of artemisinin-based combination therapy (ACT) and distribution of insecticide-treated mosquito nets (ITN) and long-lasting insecticidal nets (LLINs). In 2014, the Burkinabe National Malaria Control Programme introduced seasonal malaria chemoprevention (SMC) with amodiaquine–sulfadoxine–pyrimethamine (AQ–SP). This strategy targets children aged 3–59 months, excluding those with known allergies to sulfonamides or AQ, those who received a dose of AQ or SP within the past month, those with known HIV-positive status and under cotrimoxazole treatment, and those severely ill or experiencing a presumptive malaria episode [2].

A concern with the implementation of SMC is the absence of malaria infection screening using available diagnostic methods before the treatment of eligible children. Studies have demonstrated that in malaria endemic countries, a large proportion of malaria infections are asymptomatic [3, 4]. While guidelines do not currently recommend the treatment of asymptomatic parasitaemia with artemisinin-based combinations as is recommended for symptomatic cases, the lack of screening for malaria infection during SMC results in some number of individuals receiving AQ–SP as “treatment” for asymptomatic malaria [1]. Moreover, while the efficacy of AQ–SP as a chemoprevention has been well studied, the efficacy of AQ–SP as treatment in most of these settings has not been well characterized since the roll-out of SMC [5–7].

Therapeutic drug concentrations are influenced by pharmacokinetics, host immunity, and parasite susceptibility to the drug. Amodiaquine is metabolized to its primary active metabolite N-desethylamodiaquine (DEAQ) by the cytochrome P450 enzyme (CYP) 2C8. Two of the more common CYP2C8 genetic variants have been described to have altered drug metabolism, including the impaired conversion of amodiaquine to DEAQ; CYP2C8*2, which is more prevalent in people of African descent, and CYP2C8*3, which is more prevalent in Caucasians [8]. The role of genetic variation on the metabolism of anti-malarial drugs—known as pharmacogenomics—is not well understood, but CYP2C8 variations may be partly responsible for the wide interindividual variability in plasma levels of DEAQ, possibly contributing to inconsistencies in the efficacy and safety of AQ in the region.

Drug resistance to amodiaquine is primarily mediated by a single nucleotide polymorphism (SNP) that results in a K76T substitution in the *Plasmodium falciparum chloroquine resistance transporter* (*pfcrt*) gene. Resistance is augmented by polymorphisms in another transporter gene, *pfmdr1*. Before SMC implementation in Burkina Faso, AQ resistance mediating polymorphisms have been reported in several studies [5, 9, 10]. Selection of these drug resistance-associated SNPs has been reported after several months of SMC administration in Burkina Faso [11]. Intriguingly, artemether–lumefantrine—the most widely used first-line ACT in Burkina Faso—is known to exert an opposing selective pressure in both *pfcrt* and *pfmdr1* towards wild-type SNPs [12]. Currently, it is unknown which selective pressure predominates, but transmission of these drug resistance polymorphisms is likely to be impacted by the prevalence of asymptomatic infections and treatment with AQ–SP.

The aim of this study is to report the prevalence of microscopic and submicroscopic malaria infections before and after the first round of SMC in children under five in Bama, Burkina Faso. This study also provides a more comprehensive view of the host and parasite factors that may influence SMC, specifically day 7 plasma concentrations of DEAQ, the presence of CYP2C8 genetic variants, and the prevalence of parasite drug resistance-mediating polymorphisms.

## Methods

### Study site and population

Samples for the present study were collected in Bama, Burkina Faso, a rural area located in the district of Dandé in the Kou Valley. Bama is a rice-growing area of 1200 hectares located 30 km from Bobo-Dioulasso in the southwest of Burkina Faso. The rainy season in this area extends from June to October and the dry season from November to May. The Kou Valley is a permanent source of irrigation water with two rice crops per year from July to November and from January to May. Malaria transmission is perennial with a peak during the rainy season when the density of *Anopheles gambiae* is very high with an annual entomological inoculation rate of up to 200 infective bites/person/night [13]. The medical centre in Bama covers 6 villages, with an estimated population of 23.153 individuals in 2016. The target population for our study was an estimated 4.200 children aged 3–59 months eligible for SMC.

### SMC delivery in Bama, Burkina Faso

SMC is distributed by a team of community health workers (CHW) during the rainy season corresponding to the high malaria transmission period. In 2017, SMC was distributed four times, from the end of July to the end of October, and each distribution cycle lasted 4–5 days with an interval of 1 month between administration. Drugs were delivered to eligible children using a door-to-door strategy. Drugs were crushed and mixed with sugary water to improve uptake. A complete treatment course of SMC includes a single dose of SP and three daily doses of AQ; the CHWs provide the first dose to each child under directly observed treatment (DOT). The study team observed children for 30 min following ingestion of the first dose. In all situations where the child vomited or regurgitated the drug within 30 min, a second treatment was administered. After administration of the first dose of AQ and SP, the CHWs explained to parents/guardians how to administer the second and third doses of AQ to the child at home and informed them about possible side effects.

### Participant selection and inclusion

Two weeks’ prior the delivery of the first round of SMC, 124 randomly selected households were visited by the study team. EPI-style random walk method was utilized to randomly select households with children under 5 years of age in a two-step process which involved first selecting a starting point and secondly selecting households from that point onward. The EPI method was appropriate due to lack of local census data and boundary maps. Only one child was selected per household; if multiple children met the inclusion criteria, one child was randomly selected. Households with children aged 3 to 59 months were selected to participate in the study after the parents/guardians gave informed consent. Unoccupied houses were visited a total of three times. The first malaria prevalence survey was conducted in late July 2017, within the period of distribution of the first round of SMC. When logistically feasible, CHWs were accompanied by the study team and blood smears and dried blood spots (DBS) were collected by finger-prick. Otherwise, houses were visited ± 2 days of the 1st day of SMC administration. The team returned to collect blood samples for pharmacokinetic analyses (see Quantification of DEAQ below) 7–8 days post- SMC administration. For the second malaria prevalence survey in late August 2017, 1 week before the second round of SMC, the study team visited the same households and collected information on any symptoms related to malaria, along with smears and DBS from the same children, if available for resampling.

### Preparation of blood smears and microscopy examination

Thick and thin blood films obtained from children by finger prick were air dried, stained in Giemsa 2% for 30 min, and examined by light microscopy fitted with 100 × oil immersion lens in the laboratory of IRSS. A smear was considered negative after examination of at least 100 fields. Parasite density was calculated by counting the number of asexual parasites per 200 leukocytes, assuming a leukocyte count of 8000/μL. For all positive smears, parasite species were determined using the corresponding thin smear on the same slide. Each smear was read by two experienced microscopists. Results were considered discordant if one result was negative and the other positive for malaria infection. Discordant results were resolved by a third reader.

### DNA extraction and Polymerase Chain Reaction (PCR)

Parasite DNA was extracted from DBS using Chelex-100. *Plasmodium* species were subsequently detected by nested PCR as previously described [14]. Species were determined by amplification of 18 s RNA using nested PCR with secondary primers specific to the species *Plasmodium falciparum, Plasmodium malariae, Plasmodium ovale* and *Plasmodium vivax*. For quality control, a template free control was used in all reactions and genomic DNA from laboratory strains (www.beiressource.com) were used as positive control for respective species. All positive samples by microscopy and negative by PCR were reanalysed by PCR before confirmation of the microscopy results.

### Quantification of desethylamodiaquine (DEAQ)

A total of 200 µL of capillary whole blood was collected in EDTA-containing microtubes on day 7. Samples were centrifuged at 2000×*g* for 10 min and the plasma was then transferred to cryovials and stored at − 80 °C. DEAQ plasma concentrations were quantified by reverse phase liquid chromatography and detection with an AB Sciex API 5000 tandem mass spectrometer (LC–MS-MS) in turbo-ion spray-positive mode by North East BioLab (Hamden, CT). ^2^H^5^N-Desethylamodiaquine was used as an internal standard and the total assay coefficient of variation were < 7%. The lower limit of quantification (LLOQ) of DEAQ was 0.34 ng/mL.

### CYP2C8 genotyping

Genotyping for two nonsynonymous CYP2C8 variants was performed on DNA extracted from DBS using the QIAamp DNA Mini Kit (Qiagen). CYP2C8*2 (805A > T) and CYP2C8*3 (416G > A and 1196A > G) alleles were determined using a TaqMan Drug Metabolism Genotyping Assay (assay ID C_30634034_10 and C_25625794_10, respectively). PCR was performed in a 25 µL reaction with 12.5 µL of TaqMan Universal PCR Master Mix, 1.25 µL of the drug Metabolism Genotyping Assay Mix, and at least 3 ng of DNA template, per the manufacturer’s instructions. Quality control was maintained with no template controls with every reaction, along with previously determined wild type, heterozygous, or homozygous CYP2C8*2 or *3 allele positive controls (from K. Kidd Lab, Yale University) [15].

### Genotyping of drug resistance markers

Genotyping was performed on parasite DNA extracted from DBS using the QIAamp DNA Mini Kit (Qiagen). Nested PCR was performed to amplify amodiaquine resistance-associated alleles for *pfcrt* and *pfmdr1*. Amplicons were used for a ligase detection reaction (LDR) which contained an allele-specific and conserved sequence primer [16]. Allele-specific primers contained a 5′ nucleotide sequence unique to a MagPlex bead tagged with a complementary sequence. The 3′ end of the allele-specific primer corresponded to a particular drug resistance polymorphism: *pfcrt* K76T or *pfmdr1* N86Y. Conserved sequence primers were modified by 5′ phosphorylation and 3′ biotinylation. After primer ligation, LDR reaction products were hybridized to their respective MagPlex beads in 1.5 TMAC buffer (3 M tetramethylammonium chloride, 50 mM Tris–HCl pH 8, 3 mM EDTA, and 0.1% N-lauroylsarcosine sodium). Fluorescent labeling was performed with a 1:50 dilution of streptavidin-R-phycoerythrin. Drug resistance polymorphisms were determined by measuring fluorescent intensity using xPonent software (Luminex) on a Bio-Plex 200 instrument (Bio-Rad). Genotyping was attempted for SP-associated mutations, but due to a low success rate, data is not included.

### Statistical analysis

Data were collected with Excel and analysed by R version 3.4.0 (2017-04-21). Chi square was used to compare proportions and a *p* value of < 0.05 was considered statistically significant.

## Results

Blood samples were collected from 106 children at the start of SMC by randomly selecting one child under 5 years in each enrolled household. In the second survey, the same households were revisited and blood samples were collected from 93 children; 31 were absent from their households for three or more visits and thereby excluded. The ages of children ranged between 6 and 48 months old, with an average of 29 months. The ratio of male to female children were comparable at both time points (0.93 to 1.1).

### Malaria prevalence in first survey

DBS were collected 1 day before (16.7%) or on the day of (54.6%) SMC, while 28.7% were collected within 2 days after SMC due to field logistics. Results of *Plasmodium* infection detected by both microscopy and PCR are summarized in Table 1. 2.8% (3/106) of children were positive by microscopy and 13.2% (14/106) by nested PCR (p < 0.001). Parasites densities calculated by microscopy ranged from 200 to 9000 parasites/μL of blood. *Plasmodium falciparum* was the only species detected in positive sample by both microscopy and PCR. All microscopy positive samples were also PCR positive.

**Table 1:** Malaria infection by microscopy and PCR during the first survey

	PCR positive n/N (%)	Microscopy positive n/N (%)	PCR & Microscopy positive n/N (%)
*P. falciparum*	15/106 (14.15)	5/106 (4.71)	5/106 (4.71)
*P. vivax*	0	0	0
*P. malaria*	0	0	0
*P. ovale*	0	0	0
Total positive	15/106 (14.15)	5/106 (4.71)	5/106 (4.71)

*N* total number of samples genotyped, *n* number of microscopy or PCR positive sample

### Malaria prevalence in second survey

The proportion of malaria-positive individuals was 4.3% (4/93) by microscopy and 14.0% (13/93) by nested PCR (p = 0.0007, Table 2). None were symptomatic at the time of sample collection. Only one child was PCR-positive at both time points. Again, all positive samples by both microscopy and PCR were 100% *P*. *falciparum* single infection. For microscopy, positive samples parasite densities ranged from 960 to 62,137 parasites/μL.

**Table 2:** Malaria infection by microscopy and PCR during the second survey

	PCR positive n/N (%)	Microscopy positive n/N (%)	PCR and microscopy positive n/N (%)
*P. falciparum*	15/93 (16.1)	3/93 (3.2)	3/93 (3.2)
*P. vivax*	0	0	0
*P. malaria*	0	0	0
*P. ovale*	0	0	0
Total	15/93 (16.1)	3/93 (3.2)	3/93 (3.2)

*N* total number of samples genotyped, *n* number of microscopy or PCR positive sample

### Day 7 DEAQ drug levels after first SMC administration

**Fig. 1:**
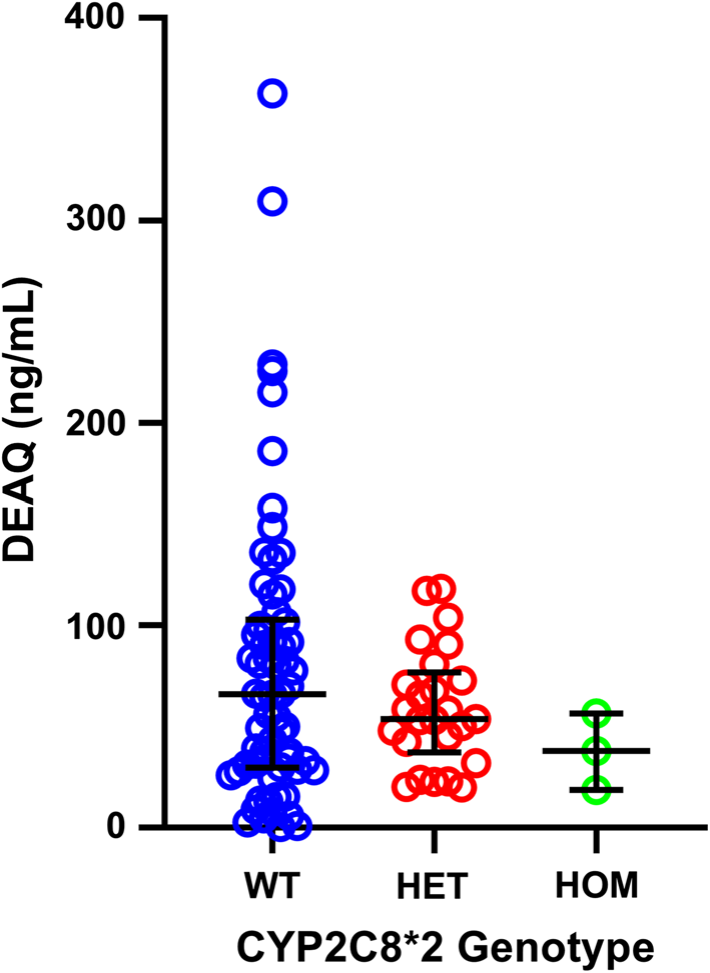
Scatterplot of Day 7 DEAQ results

A total of 105 children were sampled on Day 7 for DEAQ drug levels, the active metabolite of AQ. Drug level was quantified in 99 children, with 6 children having DEAQ levels below LLOQ. Day 7 concentrations of DEAQ ranged from 0.47 to 362.80 ng/mL with a median concentration of 56.34 ng/mL (IQR 29.62–92.61 ng/mL) (Fig. 1).

### Prevalence of CYP2C8 variants and relationship with DEAQ drug level

DNA was available from 110 children for CYP2C8 genotyping. Of those, genotyping for CYP2C8*2 variants was successful in 98.2% (108/110) of the samples. The CYP2C8*2 allelic frequency was 17.1% (10.0–24.2, 95% CI) and was found to be in Hardy–Weinberg equilibrium (χ^2^ = 0.013, *p* = 0.99). With respect to genotype, 28.7% (31/108) were heterozygous and only 2.8% (3/108) were homozygous for the CYP2C8*2 allele. Genotyping for CYP2C8*3 variants was successful in 97.3% (107/110) of the samples; however, the CYP2C8*3 allele was not detected in any of the children genotyped. Results of the genotyping are summarized in Table 3.

**Table 3:** Prevalence of CYP2C8 variants

Genotype	CYP2C8*2 N (%)	CYP2C8*3 N (%)
Wild-type	74 (68.5)	107 (100%)
Heterozygote	31 (28.7)	0
Homozygous mutant	3 (2.8)	0
Total	108	107

*N* number of cases

The relationship between DEAQ drug levels and CYP2C8*2 genotype was also assessed; however, there was no statistically significant difference in DEAQ concentration between those with homozygous wild-type alleles (median 65.93 ng/mL, IQR 30.31–101.22 ng/mL) or heterozygous CYP2C8*2 alleles (median 53.64 ng/mL, IQR 42.54–72.82 ng/mL) (*p* = 0.5216, Mann–Whitney *U* test).

### Prevalence of amodiaquine resistance markers

*Pfcrt* genotyping for the K76T SNP was successful on 85.7% (12/14) and 46.2% (6/13) of samples from the first and second surveys, respectively. The first survey showed a high proportion of mixed (58.3%, 7/12) and wild-type K76T (16.7%, 2/12), while only a single wild-type and no mixed K76T infections were detected in the second survey (16.7%, 1/6). Thus, the proportion of pure mutant infections increased from 25% (3/12) to 83.3% (5/6) between surveys. However, there was no statistically significant difference in the prevalence of polymorphisms between the first and second survey (*P* = 0.34, Fisher’s exact test), though the comparison is limited due to the small number of successfully genotyped samples in the second survey. For *pfmdr1*, genotyping for the N86Y SNP was successful on 71.4% (10/14) and 69.2% (9/13) of samples from the first and second surveys, respectively. The results showed a high prevalence of wild-type N86 alleles at both sampling time points, with only one sample positive for the 86Y polymorphism from the second survey.

## Discussion

The present study assessed the prevalence of malaria infection by microscopy and PCR in a cohort of children under 5 years old after the first round of SMC with AQ–SP in Bama, Burkina Faso. The results of this study demonstrated a relatively low but non-negligible parasitaemia in SMC eligible children after drug administration. These results further support that the majority of malaria infections are sub-microscopic [17–21]. All microscopy-positive samples were also PCR-positive and there was no discrepancy between both techniques in identifying parasite species. These results further demonstrate that a significant proportion of SMC eligible children were infected with malaria at the time they received SMC for prevention, but more notably, nearly 15% of the children were re-infected following insufficient protection from SMC, with a single case positive for malaria at both time points. This proportion of infected children following SMC represents a failure of chemoprevention and demonstrates the limitation of SMC. These failures may be explained by the high transmission intensity, drug factors that limit the duration of prophylaxis following administration. A limitation to this analysis is that approximately 30% of the samples were collected within 2 days after administration of the first dose of SMC. Although infected children are not expected to have cleared all parasites within 48 h of drug administration, this factor may have reduced the prevalence by microscopy and possibly PCR.

To probe the potential drug-related impacts, day 7 DEAQ levels were further assessed in children after SMC administration. The median day 7 DEAQ concentration in the study cohort is consistent with previous PK studies of amodiaquine and DEAQ [22, 23]. Though the influence of CYP2C8 polymorphisms on amodiaquine metabolism has been described in vitro, data on the impact of these polymorphisms in vivo is lacking. Of interest in the context of SMC, is the potential for increased toxicity associated with impaired amodiaquine metabolism; however, there has been no clear association between the CYP2C8*2 allele and amodiaquine toxicity or efficacy. In this study population CYP2C8*2 variant was found to be relatively common with a prevalence of 17.1%, comparable to the published allelic frequencies of other studies from West Africa [8, 15, 24]. To explore the potential impact of the CYP2C8*2 variant on amodiaquine metabolism, day 7 DEAQ concentrations were compared between children with homozygous wild-type, heterozygous, and homozygous CYP2C8*2 mutant alleles. While a trend towards a decrease in DEAQ concentrations could be observed from wild-type to heterozygous alleles, this decrease was not statistically significant. Children homozygous for the CYP2C8*2 allele had the lowest concentrations of DEAQ in this study but the small number of homozygous CYP2C8*2 children (n = 3) precluded any meaningful analysis. It is possible that a single wild-type and mutant allele provides enough metabolic turnover of amodiaquine to maintain therapeutic ranges of DEAQ. Caution should be used to interpret these results; given the sample size in this study of 110 children and a predicted 3:1 ratio of wild-type to CYP2C8*2 heterozygous children, the study had 80% power to detect a 33.3% decrease in DEAQ levels. It is important to note that this was an “effectiveness” study, and consistent with national SMC guidelines, only the first dose of amodiaquine was directly observed. There are supervisors in each village that perform household visits to improve programme implementation and treatment adherence, but this cannot guarantee compliance. Caregiver delay or omission of subsequent doses would impact drug concentration–time profiles, which would complicate interpretation of day 7 DEAQ levels. Whether or not homozygous/heterozygous CYP2C8*2 children are at increased risk of amodiaquine toxicity or reduced efficacy warrants consideration for further study.

As reported in other studies [11, 25, 26], a major concern with the use of SMC is an increase in the proportion of parasites carrying AQ and SP resistance-mediating polymorphisms, jeopardizing the future of malaria prevention and control. However, it is not yet well established whether the prophylactic failure of SMC is associated with emergence of resistance. Genotyping for putative AQ resistance-associated markers revealed that there was a large proportion of mixed infections during the first survey, present in more than half of genotyped samples. While the second survey showed a much higher proportion of *pfcrt* 76T mutations, associated with reduced amodiaquine sensitivity, less than half of the PCR-positive samples could be genotyped at this time point by our methods. Genotyping failure is likely attributed to the low-density of these infections, reflected in the limited number of these detected by microscopy, and also in the lack of successful SP-associated mutation typing. In contrast to *pfcrt* polymorphisms, there was limited genetic diversity at the *pfmdr1* N86Y loci, with all but one sample positive for the N86 wild-type SNP. As noted earlier, lumefantrine selects toward the wild-type N86, in opposition to the direction of selection for amodiaquine. Careful monitoring of both AQ and SP-resistance associated polymorphisms and of possible prophylactic failures of SMC should be ensured in order to understand the potential population-level impacts of the opposing selection pressure by the first-line treatment, artemether–lumefantrine, and the widespread SMC regimen, AQ–SP. If possible, future studies should encompass multiple rounds of SMC, with testing at baseline, throughout, and after SMC to assess for the selection of resistance. Finally, more in-depth pharmacokinetic studies with detailed drug resistance typing may be required to understand the relationship between AQ–SP exposure and the development of resistance.

## Conclusion

This study estimated the prevalence of malaria infection in children at two time points after treatment with the first round of seasonal chemoprophylactic AQ–SP, demonstrating a notable prevalence of low-level malaria parasitaemia in children 3 weeks after the first round of SMC. Day 7 concentrations of the active DEAQ metabolite varied widely, likely reflecting variability in adherence and possibly metabolism. The findings of this study highlight factors that may contribute to the effectiveness of SMC in children in a high transmission season.

## Data Availability

All data generated or analysed during this study are included in this published article

## Abbreviations

ACT: Artemisinin-based Combination Therapy
ARN: Acid Ribonucleic
AQ–SP: Amodiaquine–Sulfadoxine–pyrimethamine
bp: Base pair
CEIRES: *Comité d’Ethique Institutionnel pour la Recherche en Santé*
CHW: Community health workers
DBS: Dried Blood Spots
DHA-PQ: Dihydroartemisinin–piperaquine
DNA: Deoxyribonucleic acid
DOT: Directly observed treatment
EIR: Entomological inoculation rate
g/dl: Gram/deciliter
IRSS-DRO: Institut de Recherche en Sciences de la Santé- Direction Régionale de l’Ouest
LLIN: Long-lasting insecticidal nets
PCR: Polymerase Chain Reaction
RDT: Rapid Diagnostic Test
SMC: Seasonal malaria chemoprevention
UV: Ultra-violet
